# Supplementary material for: Novel Driver Strength Index highlights important cancer genes in TCGA PanCanAtlas patients
Source: PeerJ. 2022 Aug 11;10:e13860. doi: 10.7717/peerj.13860 (PMC9375969; doi:10.7717/peerj.13860)
Supplement: Supplemental Information 2 [file peerj-10-13860-s002.zip › COHORTS/genes plots/cohorts/2021_8_16_14_9_distribution_cohorts_ESCA.pdf]

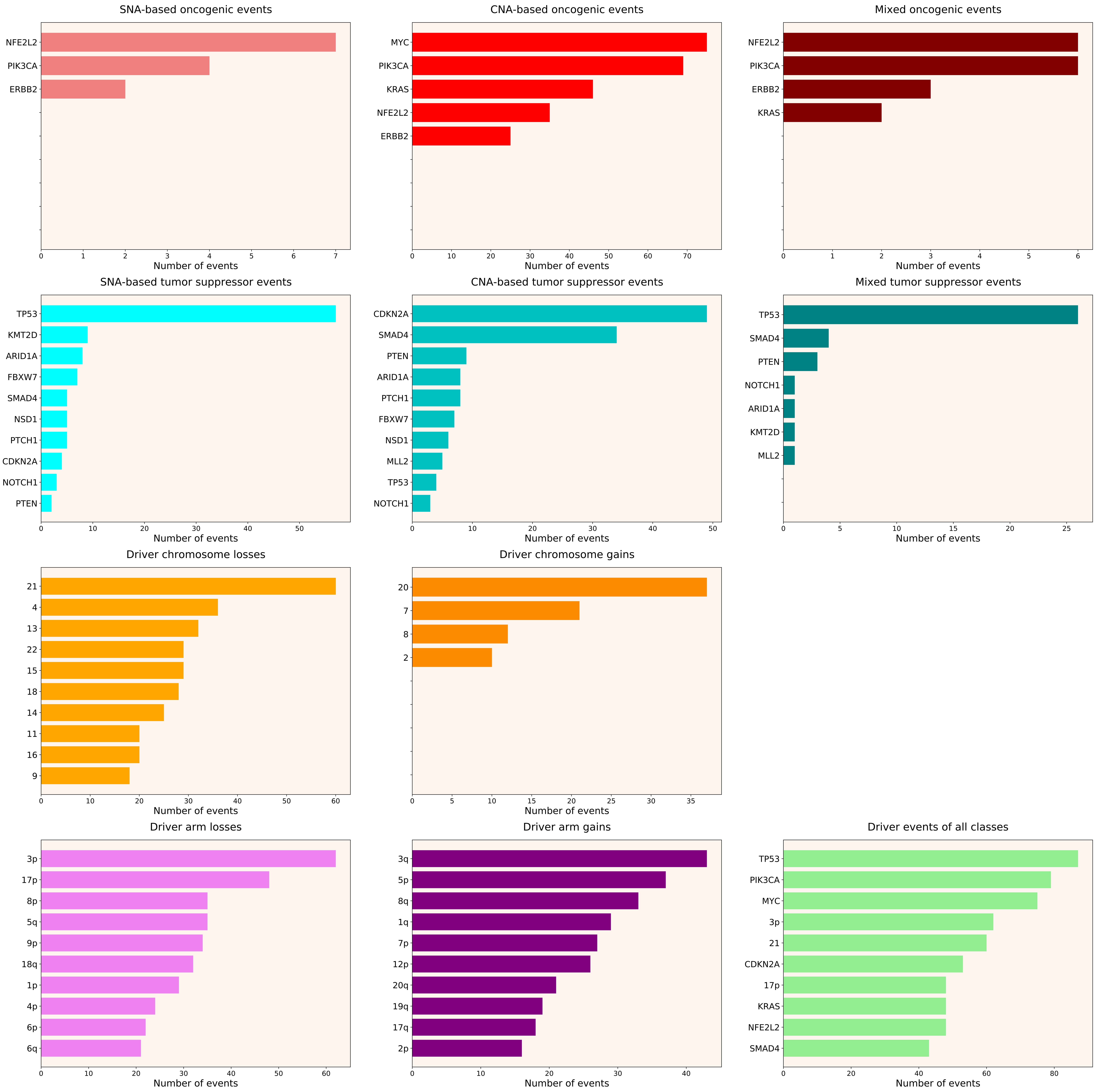

SNA-based oncogenic events
NFE2L2
PIK3CA
ERBB2
0 1 2 3 4 5 6 7
Number of events
CNA-based oncogenic events
MYC
PIK3CA
KRAS
NFE2L2
ERBB2
0 10 20 30 40 50 60 70
Number of events
Mixed oncogenic events
NFE2L2
PIK3CA
ERBB2
KRAS
0 1 2 3 4 5 6
Number of events
SNA-based tumor suppressor events
TP53
KMT2D
ARID1A
FBXW7
SMAD4
NSD1
PTCH1
CDKN2A
NOTCH1
PTEN
0 10 20 30 40 50
Number of events
CNA-based tumor suppressor events
CDKN2A
SMAD4
PTEN
ARID1A
PTCH1
FBXW7
NSD1
MLL2
TP53
NOTCH1
0 10 20 30 40 50
Number of events
Mixed tumor suppressor events
TP53
SMAD4
PTEN
NOTCH1
ARID1A
KMT2D
MLL2
0 5 10 15 20 25
Number of events
Driver chromosome losses
21
4
13
22
15
18
14
11
16
9
0 10 20 30 40 50 60
Number of events
Driver chromosome gains
20
7
8
2
0 5 10 15 20 25 30 35
Number of events
Driver arm losses
3p
17p
8p
5q
9p
18q
1p
4p
6p
6q
0 10 20 30 40 50 60
Number of events
Driver arm gains
3q
5p
8q
1q
7p
12p
20q
19q
17q
2p
0 10 20 30 40
Number of events
Driver events of all classes
TP53
PIK3CA
MYC
3p
21
CDKN2A
17p
KRAS
NFE2L2
SMAD4
0 20 40 60 80
Number of events
